# Cardio-Metabolic Risk in Adults Born Preterm: A Narrative Review

**DOI:** 10.3390/jcm15010256

**Published:** 2025-12-29

**Authors:** Benjamim Ficial, Leonardo Gottin, Claudio Maffeis

**Affiliations:** 1 Neonatal Intensive Care Unit, University and Hospital Trust of Verona, P. le A. Stefani 1, 37126 Verona, Italy; 2 Radboud Institute for Health Sciences, Amalia Children’s Hospital, Division of Neonatology, Department of Pediatrics, Radboud University Medical Center, Geert Grooteplein Zuid 10, 804, 6525 GA Nijmegen, The Netherlands; 3 Intensive Care Unit, Department of Surgery, Dentistry, Pediatrics, and Gynecology, University and Hospital Trust of Verona, P. le A. Stefani 1, 37126 Verona, Italy; 4 Section of Pediatric Diabetes and Metabolism, Department of Surgery, Dentistry, Pediatrics, and Gynecology, University of Verona, 37126 Verona, Italy

**Keywords:** adult borne preterm, obesity, non-communicable diseases, hypertension, preterm, diabetes, stroke, metabolic syndrome

## Abstract

Preterm birth has evolved from being an acute neonatal challenge to a lifelong health determinant, as advances in neonatal care have markedly improved the survival of very and extremely preterm infants. This narrative review synthesizes epidemiological and mechanistic evidence linking preterm birth with heightened cardiometabolic risk across the life course. In adulthood, individuals born preterm demonstrate increased rates of heart failure, ischemic heart disease, stroke, atrial fibrillation, and diabetes. Beneath these overt clinical outcomes lies a distinct phenotype characterized by increased adiposity, insulin resistance, dyslipidemia, hypertension, and atypical growth trajectories, with rapid catch-up growth amplifying long-term risk. Mechanistic pathways highlight adipose tissue maldevelopment, predisposing to metabolic syndrome, alongside cardiac maldevelopment with reduced ventricular size, impaired diastolic function, and diminished exercise capacity. Furthermore, vascular growth arrest, impaired elastin synthesis, and nephron deficiency contribute to sustained elevations in blood pressure, establishing an early substrate for hypertension and cardiovascular remodeling. These alterations reflect the developmental origins of health and disease, whereby early-life disruption of growth and maturation exerts lasting effects on organ structure and function. Collectively, the evidence identifies adults born preterm as a growing yet under-recognized patient population with a unique clinical and biochemical profile and accelerated vulnerability to non-communicable diseases. Greater awareness among pediatric and adult physicians, structured transition of care, and targeted prevention strategies are urgently needed to mitigate early cardiometabolic morbidity and optimize long-term health outcomes in this high-risk group.

## 1. Introduction

Prematurity, defined as birth before 37 weeks of gestation, remains a major global public health concern [1]. Each year, approximately 13.4 million newborns are delivered preterm, accounting for about 1 in 10 live births worldwide [2]. Alarmingly, nearly one million of these infants die annually due to complications associated with prematurity [3]. Preterm birth is responsible for approximately 40% of all neonatal deaths, making it the leading cause of death in children under five years of age globally, as well as the most common cause of perinatal mortality [4,5].

While advances in neonatal care have markedly improved survival—particularly among very low birth weight (VLBW) and extremely preterm infants—there is growing recognition of the long-term health implications for this population [6]. Increasing evidence indicates that individuals born preterm are at heightened risk for a range of non-communicable diseases (NCDs) later in life, notably cardiovascular and metabolic disorders such as ischemic heart disease, heart failure, hypertension, diabetes, and obesity [7,8,9,10].

At the same time, the world is witnessing a global surge in overweight and obesity, accompanied by a parallel increase in NCDs—notably cardiovascular and cerebrovascular disorders [11]. Within this broader epidemic, a critical concept arises in the context of personalized medicine: amidst the general population affected by obesity, metabolic syndrome, and cardiovascular diseases, there exists a distinct and particularly vulnerable subgroup—adults born preterm [12].

This population presents with novel clinical phenotypes, characterized by early-onset cardiovascular and metabolic dysfunction, atypical developmental trajectories, and unique constellations of risk factors. These individuals do not simply exhibit an accelerated version of adult-onset disease, but rather may represent a distinct pathophysiological category, demanding specific clinical attention, focused research efforts, and individualized prevention and treatment strategies [13].

This narrative review explores the current understanding of cardiometabolic risk in adults born preterm, examining epidemiological trends, mechanistic pathways, and clinical implications, with an emphasis on the need for targeted prevention and management approaches in this growing and under-recognized group.

## 2. Adult Born Preterm and Cardiovascular Disease: The Tip of the Iceberg

Recent evidence has increasingly highlighted the long-term cardiovascular consequences in adulthood of preterm birth, most prominently heart failure, ischemic heart disease, stroke, and type 2 diabetes—conditions that represent the “tip of the iceberg.” [7]. Main studies are summarized in Table 1.

### 2.1. Adults Born Preterm and Heart Failure

A large registry-based cohort study in Sweden, involving over 2.6 million individuals, revealed a significant association between preterm birth and the development of heart failure (HF) unrelated to ischemic heart disease. Importantly, this association persisted even after adjusting for birth weight and other potential confounders. The risk of HF was inversely related to gestational age (GA): individuals born before 28 weeks of gestation had a 17-fold increased risk, while those born very preterm (28–31 weeks) faced a more than 3-fold higher risk compared to those born at term. Notably, the median age at HF diagnosis was just 16.5 years (interquartile range: 5.2 to 19.7 years), underscoring the early onset and severity of cardiovascular complications in this vulnerable population [14]. Notably, the authors’ exclusion of cases attributable to ischemic heart disease suggests that the primary driver of the increased risk of heart failure is developmental alteration or maldevelopment, rather than acquired vascular pathology. This finding underscores the profound influence of early life conditions on long-term cardiovascular health, potentially laying the foundation for premature cardiovascular aging and the early onset of chronic disease [24].

### 2.2. Adults Born Preterm and Ischemic Heart Disease

Recent research has begun to clarify the association between preterm birth and ischemic heart disease (IHD). While earlier studies reported little to no link [15]—likely due to survivor bias or limited follow-up into adulthood—a large national cohort study from Sweden has provided important new insights. Tracking more than 2 million individuals up to age 43, the study found that GA at birth was inversely associated with the risk of IHD in adulthood. After adjusting for perinatal, maternal, and familial factors, preterm birth was associated with a 53% increased risk of IHD (95% CI, 20–94%), while early-term birth conferred a 19% increased risk (95% CI, 1–40%) between ages 30 and 43. Notably, preterm-born men showed the highest incidence of IHD, whereas preterm-born women had the highest relative risk—likely reflecting lower baseline rates of IHD among women born at term [16].

### 2.3. Adults Born Preterm and Atrial Fibrillation

Lastly, recent evidence has identified a link between preterm birth, large for GA, and the risk of atrial fibrillation (AF) in a large population-based study. Although AF primarily affects middle-aged and older adults, its incidence among children and young adults has modestly increased in recent decades, often without identifiable underlying causes. Emerging evidence suggests that preterm birth may be an important contributing factor to this early-onset AF risk [17].

### 2.4. Adults Born Preterm and Cerebrovascular Disease

An expanding body of evidence indicates a significant association between preterm birth and the risk of cerebrovascular disease, particularly stroke [20,25,26]. The most recent and largest population-based study from Sweden, including over 2.1 million singletons who were followed up for first-time stroke, provides compelling evidence that preterm birth is associated with an increased risk of stroke in adulthood (between 18 and 43 years) [18]. The study demonstrated that preterm birth (<37 weeks) was associated with a significantly increased risk of first-time stroke (adjusted hazard ratio [HR] 1.26, 95% CI 1.12–1.43. When stratified by gestational age, a clear dose–response relationship emerged: the lower the gestational age, the higher the stroke risk. Specifically, early preterm birth (22–33 weeks) was associated with a markedly increased risk (HR 1.42, 95% CI 1.11–1.81), whereas late preterm birth (34–36 weeks) was also linked to elevated risk, though of smaller magnitude (HR 1.22, 95% CI 1.06–1.40). Both ischemic and hemorrhagic stroke risks were increased, with effect sizes of similar magnitude. These associations were observed consistently for both hemorrhagic and ischemic stroke, with similar magnitudes of effect [18].

Importantly, the study also performed extensive analyses to account for potential confounding. In co-sibling models, which control for unmeasured shared familial (genetic and environmental) factors, the associations were attenuated but remained elevated, indicating that preterm birth itself is an independent determinant of stroke risk.

The findings further suggest that the association between preterm birth and stroke is stronger in more recent birth cohorts. A likely explanation is survivor bias: in earlier decades, neonatal mortality among the most vulnerable preterm infants was very high, meaning that only the healthiest survivors reached adulthood. With advances in neonatal care, survival has improved, allowing a broader range of preterm infants—including those with higher long-term cardiometabolic vulnerability—to reach adulthood. This shift likely explains why earlier studies did not consistently identify an association between preterm birth and stroke [15,19], whereas this large and contemporary study was able to demonstrate a robust link [18].

Taken together, these results highlight preterm birth as an independent and lifelong risk factor for stroke, with increasing public health relevance as survival of preterm infants continues to improve worldwide [27].

### 2.5. Adults Born Preterm and Diabetes

Growing evidence indicates that preterm birth is linked to elevated risks of both type 1 and type 2 diabetes, beginning in childhood and extending into early to mid-adulthood. A large Swedish cohort study involving over 4 million individuals found that preterm birth (≤36 weeks of GA), was associated with a 1.2-fold increased risk of type 1 diabetes and a 1.3-fold increased risk of type 2 diabetes before age 18. Between ages 18 and 43, these risks rose to 1.2-fold and 1.5-fold, respectively. The association with type 2 diabetes was notably stronger among females. Additionally, births at early term (37–38 weeks) were also linked to a modestly increased risk for both types of diabetes across the lifespan. Co-sibling analyses suggested that shared familial genetic or environmental factors only partially accounted for these associations [21].

Previous studies have consistently reported associations between preterm birth and increased risk of both type 1 and type 2 diabetes during childhood [22,28]. These findings have been reinforced by a large meta-analysis, which demonstrated that individuals born preterm had a significantly higher risk of developing type 1 diabetes compared to those born at term (OR = 1.18; 95% CI: 1.11–1.25). Similarly, preterm birth was associated with a markedly elevated risk of type 2 diabetes (OR = 1.51; 95% CI: 1.32–1.72) [23].

## 3. Adult Born Preterm and Cardiovascular Disease: What’s Below the Waterline?

**Figure 1:**
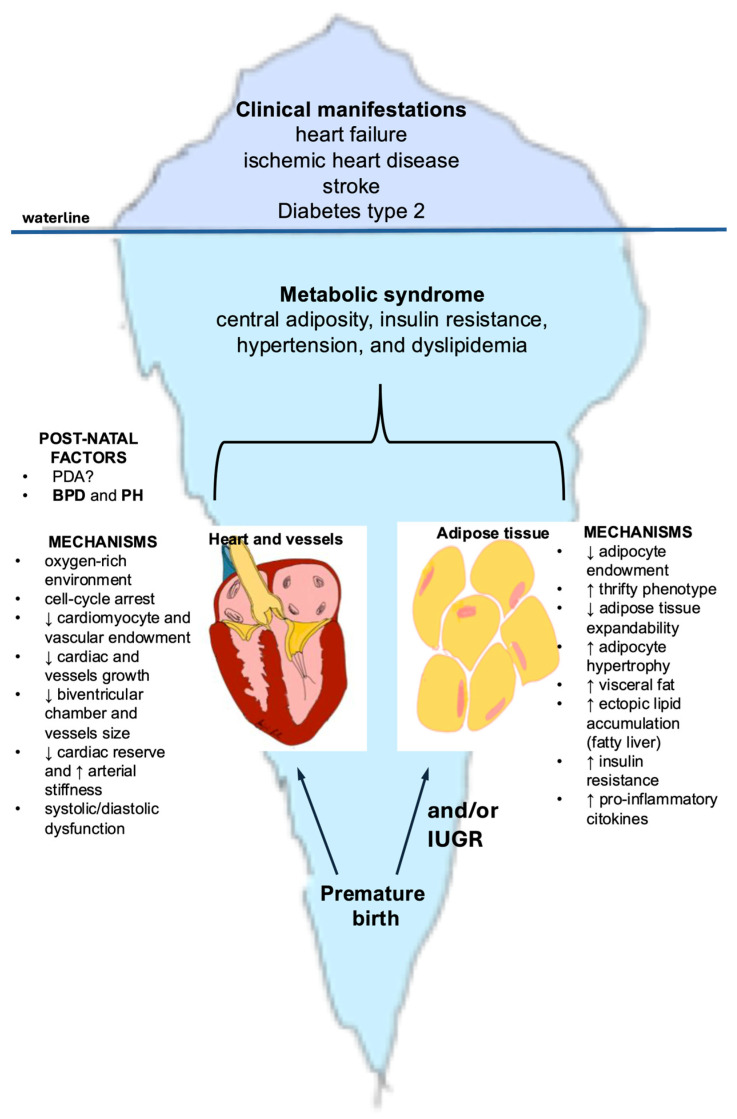
Iceberg model illustrating developmental mechanisms underlying cardiometabolic disease in adults born preterm. The portion above the waterline represents the clinical manifestations that emerge in adolescence and adulthood among individuals born preterm, including heart failure, ischemic heart disease, stroke, and type 2 diabetes. The larger submerged portion illustrates the hidden developmental origins, beginning with preterm birth and involving two major mechanistic pathways: cardiac maldevelopment (reduced cardiomyocyte and vascular endowment, smaller ventricular chambers and vessels, diminished cardiac reserve, increased arterial stiffness) and adipose tissue dysregulation (reduced adipocyte number, limited expandability, increased visceral and ectopic fat, insulin resistance, and pro-inflammatory signaling). Together, these early-life alterations contribute to the emergence of the metabolic syndrome, which forms the biological basis for the later cardiometabolic burden. Abbreviations: BPD: bronchopulmonary displasia; IUGR = intrauterine growth restriction; PDA = patent ductus arteriosus; PH = pulmonary hypertension.

In adulthood, individuals born preterm exhibit an increased burden of overt cardiometabolic diseases, most prominently heart failure, ischemic heart disease, stroke, and type 2 diabetes—conditions that represent the “tip of the iceberg” [29]. Beneath these clinical manifestations lies a common underlying substrate: the metabolic syndrome, a cluster of interrelated risk factors that includes central adiposity, insulin resistance, hypertension, and dyslipidemia [30]. Importantly, in those born preterm, the clustering of cardiometabolic risk factors, defined as metabolic syndrome, often presents with peculiar features, including increased body fat, reduced muscle mass, and unique trajectories of blood pressure and impaired glucose tolerance. This distinct clustering of risk factors may constitute the shared pathophysiological denominator that mediates and amplifies the progression from preterm birth to overt cardiometabolic disease [31]. Figure 1 depicts the developmental mechanisms through which preterm birth predisposes to cardiometabolic disease in adulthood.

### 3.1. Adults Born Preterm and Obesity

A recent meta-analysis investigated the risk of childhood obesity at age 6 to 16 following preterm birth compared with term birth. Childhood obesity was defined as a body mass index (BMI) ≥ 95th percentile according to age- and sex-specific growth standards. The pooled sample included 156,439 children. The analysis demonstrated that preterm birth was associated with a significantly increased risk of childhood obesity, with an odds ratio (OR) of 1.19 (95% CI, 1.13–1.26) [32]. Childhood obesity substantially increases the likelihood of persisting obesity into adulthood and is therefore a key driver of NCDs, including type 2 diabetes, and cardiovascular disease [33,34,35].

Moreover, the meta-analysis evaluated the impact of accelerated weight gain during the first two years of life on the subsequent risk of obesity at 8–11 years of age. The pooled results of 2129 preterm participants indicated that accelerated weight gain was significantly associated with an increased likelihood of childhood obesity (OR 1.87; 95% CI, 1.57–2.23) [32].

Preterm birth, with or without fetal growth restriction, exposes infants to serious complications during the prenatal and early postnatal periods and can have a profound impact on long-term health. This concept, known as the Barker hypothesis—also referred to as the Developmental Origins of Health and Disease (DOHAD) hypothesis—proposes that early-life exposure to unfavorable environments can “reprogram” fetal structure, function, and metabolism. Such programming increases the individual’s risk of developing NCDs later in life [36].

This is particularly relevant for extremely and very preterm infants who experience growth restriction both in utero and ex utero. These children can develop the “thrifty phenotype”, characterized by adaptive changes such as altered energy metabolism and epigenetic modifications (e.g., DNA methylation) [37,38]. During early childhood, they typically undergo rapid catch-up growth, a process that, as mentioned above, is linked to obesity and long-term metabolic consequences.

According to a recent meta-analysis, preterm infants, despite a lower weight, have already a relatively greater amount of fat tissue at term-equivalent age compared with term infants at birth [39]. Moreover, rapid weight gain, which usually occurs during infancy in these children, appears to be associated with an increase in fat mass rather than lean body mass [40]. Preterm infants also tend to accumulate more visceral adipose tissue. For example, Uthaya et al. reported that preterm infants demonstrated a reduction in subcutaneous adipose tissue accompanied by a marked increase in intra-abdominal adipose tissue [41]. Similarly, several studies have shown that preterm birth is associated with greater visceral adiposity in both adolescents and adults [42,43]. Furthermore, survivors born at extremely low birth weight exhibit, in early adulthood, a higher proportion of hepatic and pancreatic fat compared to individuals born at normal birth weight [44].

On the other hand, preterm infants often present with short stature and decreased lean body mass, which in turn results in lower resting energy expenditure. In young adults born with very low birth weight, this reduced energy expenditure may further exacerbate metabolic risks and prove detrimental in the long term [45].

Insights from the Boston Birth Cohort study, a longitudinal study involving more than 3000 children followed from infancy to 18 years of age, identified four distinct growth trajectories from birth to age 10 years to help the clinicians to identify children at risk of overweight/obesity:

- Early-onset overweight/obesity: characterized by high body mass index (BMI) beginning in early childhood, which was maintained throughout childhood.
- Late-onset overweight/obesity: marked by rapid catch-up growth in early infancy, with BMI acceleration starting around age 3 years and progressing to high BMI in later childhood (5–6 years). This trajectory has been closely linked to preterm birth.
- Normal-stable: children maintained a consistently normal BMI throughout the study period.
- Low-stable: children demonstrated a persistently low BMI across childhood [46].

Several studies corroborate the findings of the Boston Birth Cohort study, showing that accelerated weight gain between 2.5 and 6 years of age is strongly associated with increased BMI and a heightened risk of cardiovascular complications in early adulthood [47,48].

### 3.2. The Missing Link Between Premature Birth, Obesity, and Metabolic Sequelae

Preterm birth is associated with maldevelopment of adipose tissue [49]. The total number of adipocytes is largely determined during critical developmental windows—particularly in the second half of fetal life for term infants and up to term-equivalent age for preterm infants [50]. Infants with low birth weight (LBW), whether preterm or term with intrauterine growth restriction (IUGR), are often exposed to malnutrition during these sensitive periods. Consequently, they may develop a smaller lifelong adipocyte pool [49].

Adipocytes—predominantly in subcutaneous adipose tissue—function as a metabolic buffer, protecting other organs from lipotoxicity, in line with the “adipose tissue expandability” hypothesis [51]. In postnatal life, when exposed to nutrient overload, these infants with thrifty phenotype easily gain excessive energy. The reduced adipocyte pool imposes a disproportionate energy storage burden on individual cells. This leads to adipocyte hypertrophy, increased visceral fat accumulation—which exhibits a less favorable adipokine and inflammatory profile—and stromal hyperproliferation with macrophage infiltration [52,53]. All these factors contribute to insulin resistance [54].

Inflammatory pathways play a central role. For example, interleukin-6—consistently elevated in obese children and adolescents—inhibits insulin signaling across insulin-sensitive tissues. In parallel, there is a reduction in adiponectin, an insulin-sensitizing hormone. Together, these alterations exacerbate insulin resistance [55].

Adipocyte hypertrophy also promotes ectopic lipid accumulation in non-adipocyte cells, causing lipotoxic insults, apoptosis and inflammation, further contributing to insulin resistance. Of particular importance is the fat accumulation in the liver, leading to the development of non-alcoholic fatty liver disease (NAFLD), which has been recently defined as the Metabolic Disfunction-Associated Steatotic Liver Disease (MASLD) [56]. On the one hand, insulin resistance promotes hepatic triglyceride synthesis through excessive uptake of circulating free fatty acids as well as insulin-dependent de novo lipogenesis via activation of SREBP-1. On the other hand, MASLD amplifies both local and systemic inflammation and worsens insulin resistance through an altered hepatic secretory profile, notably including excess fetuin B [57].

These mechanisms contribute to the development of metabolic diseases in late adolescence and adulthood [47]. A growing body of evidence confirms that individuals born preterm exhibit an unfavorable cardiometabolic profile—often referred to as metabolic syndrome—already evident in early adolescence and increasingly prevalent in adulthood [58,59,60,61]. This profile is characterized by increased adiposity, dyslipidemia (elevated triglycerides, reduced HDL and increased LDL cholesterol), impaired fasting glucose, and elevated blood pressure [62]. Although these alterations may initially present with little or no clinical manifestation—representing what lies below the waterline of the iceberg—over time they may progress and ultimately manifest as type 2 diabetes and cardiovascular disease, which correspond to the visible tip of the iceberg [9].

Finally, recent evidence indicates that MASLD is independently associated with subclinical myocardial dysfunction, even in the absence of overt cardiovascular disease [63]. Speckle tracking echocardiography (STE) has revealed subtle impairments in left ventricular mechanics, particularly reduced global longitudinal strain, which may not be detected by conventional echocardiographic assessment, supporting the presence of early myocardial involvement related to MASLD [64]. As one of the components of the metabolic syndrome, MASLD may represent a key mechanistic pathway linking early-life adversity to increased cardiovascular risk in adulthood.

### 3.3. Cardiac Maldevelopment and Remodeling

There is increasing recognition of the heightened burden of cardiovascular manifestations—including heart failure and ischemic heart disease—among adults born preterm [65]. This population exhibits distinctive characteristics that confer an elevated susceptibility to cardiovascular risk from an early age [12]. As outlined previously, individuals born preterm are more likely to develop metabolic syndrome during adolescence and early adulthood, thereby accumulating a cluster of risk factors that predispose to cardiovascular disease [58].

Furthermore, preterm birth has a profound impact on cardiac development, leading to structural and functional alterations that often persist into adult life [24].

The third trimester represents a critical window of rapid cardiac growth, primarily driven by cardiomyocyte proliferation [66]. Preterm birth disrupts this process, as the premature exposure to an oxygen-rich extrauterine environment promotes the generation of reactive oxygen species (ROS), leading to cell-cycle arrest of cardiomyocytes and impaired proliferation of cardiomyocyte precursors. Consequently, the heart is forced into an early transition from hyperplastic to hypertrophic growth, which may permanently constrain cardiomyocyte endowment and compromise long-term cardiac reserve [67].

The arrest in cardiac development observed after preterm birth is reflected in smaller left ventricular end-diastolic volumes, reduced internal LV cavity dimensions, and shorter LV lengths: these findings have been consistently demonstrated across multiple studies using echocardiography and magnetic resonance imaging in adolescents and young adults born preterm [68,69,70,71]. In contrast, evidence regarding LV wall thickness and LV mass remains conflicting, with some studies reporting increased values and others reporting reductions [68,70,71]. It is plausible that both phenotypes coexist: at one end of the spectrum, individuals born extremely preterm may have a reduced cardiomyocyte endowment, such that even with compensatory hypertrophy, overall LV mass does not increase. At the other end, those born moderately or late preterm may retain a larger cardiomyocyte pool and undergo hypertrophic remodeling, resulting in increased LV mass compared with term-born controls [66]. Moreover, the heart is likely to undergo further remodeling in the presence of additional risk factors commonly associated with preterm birth, such as pulmonary and systemic hypertension or obesity [66,72].

Importantly, these structural alterations translate into impaired myocardial function and reduced cardiac reserve. Although left ventricular (LV) ejection fraction often appears preserved at rest, subtle abnormalities such as diastolic dysfunction are frequently detectable [68,70,73]. Under exercise stress, preterm-born young adults demonstrate a blunted augmentation of LV systolic function, impaired right ventricular (RV) contractile reserve, and reduced peak oxygen consumption, as shown by both invasive and non-invasive cardiopulmonary exercise testing [74]. Furthermore, bronchopulmonary dysplasia—the most common respiratory sequela of preterm birth—is associated with pulmonary vascular disease and pulmonary hypertension, conditions that can further exacerbate RV dysfunction and precipitate right heart failure [75]. Collectively, the evidence supports the concept that preterm birth results in persistent cardiac remodeling, diminished functional reserve, and heightened vulnerability to cardiovascular disease across the life course.

### 3.4. Adults Born Preterm and Hypertension

In a recent meta-analysis including more than 18,000 preterm and 294,000 term-born adults, preterm birth was consistently associated with higher blood pressure. Adults born preterm had significantly elevated systolic and diastolic blood pressure, both in office and 24 h measurements, compared with controls. Sex is likely to be an important contributor, as women born preterm demonstrated comparatively greater increases in blood pressure than men [76]. These findings indicate that prematurity confers a sustained increase in blood pressure into adult life, representing an important component of long-term cardiovascular risk.

Two main pathophysiological mechanisms appear to underlie the increased risk of hypertension in individuals born preterm. First, preterm birth interrupts vascular development, as the transition to extrauterine life occurs before the completion of critical processes such as angiogenesis, elastin synthesis, and vascular tree maturation. Consequently, preterm-born individuals often present with smaller aortic luminal diameters, narrower conduit arteries, reduced systemic vascular growth, and increased arterial stiffness. These vascular changes establish an early substrate for elevated blood pressure [77,78]. Second, impaired nephrogenesis leads to a reduced nephron endowment and smaller kidney size. This deficit promotes glomerular hyperfiltration, sodium and fluid retention, expansion of extracellular fluid volume, and ultimately higher arterial pressure [79,80]. Additional contributors include heightened sympathetic nervous system activity and hypothalamic–pituitary–adrenal axis dysregulation [66]. Finally, perinatal complications and interventions—such as accelerated postnatal weight gain, parenteral nutrition, and corticosteroid exposure—may further exacerbate vascular stiffness and long-term cardiovascular remodeling [66].

Collectively, these mechanisms suggest that preterm birth results in restricted vascular growth, stiffer and narrower arteries, impaired renal reserve, and altered neurohormonal regulation, all converging to increase the risk of hypertension and cardiovascular disease across the life course.

### 3.5. Limitations

This narrative review has several limitations that should be acknowledged. First, although prematurity is discussed as a major determinant of long-term cardio-metabolic risk, we did not specifically review the contribution of perinatal interventions and postnatal exposures, such as antenatal corticosteroid administration, etc. We acknowledge that cardio-metabolic risk in individuals born preterm is multifactorial and that these perinatal exposures may substantially modify long-term cardiovascular and metabolic trajectories. A comprehensive synthesis of these mechanisms was considered beyond the scope of the present review, and readers are therefore referred to other dedicated reviews addressing this topic [81].

## 4. Conclusions

Advances in neonatal care have markedly improved the survival of preterm infants, and the first generations of individuals born prematurely with extremely low birth weight have reached adulthood. This population carries an elevated risk of cardiometabolic sequelae rooted in disrupted fetal development and early postnatal exposures, resulting in a distinct clinical and biochemical phenotype. Greater awareness is required among neonatologists and pediatricians to ensure long-term surveillance, as well as among adult physicians who will increasingly care for this new patient group at risk of developing NCDs in early adulthood. Particular emphasis should be placed on the transition of care from pediatric to adult services, to ensure continuity of follow-up and the implementation of timely preventive strategies.

## Figures and Tables

**Table 1:** Summary of Main Studies on Adults Born Preterm and Cardiometabolic Outcomes.

Author(s)	Year	Study Type	Main Findings
Adults Born Preterm and Heart Failure (HF)			
Carr H. et al. [14]	2017	Swedish Nationwide Cohort	Preterm birth strongly linked to HF independent of ischemic disease; inverse GA–risk relation; extremely preterm → 17-fold risk; very preterm → >3-fold risk; median diagnosis age 16.5 yrs.
Adults Born Preterm and Ischemic Heart Disease (IHD)			
Kajantie E. et al. [15]	2015	The Helsinki Birth Cohort Study	Little/no association, likely due to survivor bias or limited follow-up.
Crump C. et al. [16]	2019	Swedish National Cohort Study	GA inversely associated with IHD; preterm → 53% increased risk; early-term → 19% increased risk; highest incidence in men, highest relative risk in women.
Adults Born Preterm and Atrial Fibrillation (AF)			
Yang F. et al. [17]	2023	Population-based study	Preterm birth and large-for-GA associated with increased risk of early-onset AF.
Adults Born Preterm and Cerebrovascular Disease (CVD)			
Crump C. et al. [18]	2021	National Cohort and Cosibling Stud	Preterm (<37 wks) → hazard ratio (HR) 1.26 for first stroke; early preterm HR 1.42; late preterm HR 1.22; both ischemic and hemorrhagic stroke increased; associations persist in co-sibling analyses
Osmond C. et al. [19]	2007	Helsinki birth cohort study	Earlier study. No consistent association—likely survivor bias.
Ueda P. et al. [20]	2014	Population-based Swedish cohort study	General evidence linking preterm birth to elevated adult stroke risk.
Adults Born Preterm and Diabetes			
Crump C. et al. [21]	2020	Swedish Nationwide Cohort	Preterm birth increases T1D and T2D risk across lifespan; before 18: T1D ↑1.2×, T2D ↑1.3×; ages 18–43: T1D ↑1.2×, T2D ↑1.5× (higher in females).
Kajantie E. et al. [22]	2010	The Helsinki birth cohort study.	Consistent association between preterm birth and higher diabetes risk in childhood.
Li S. et al. [23]	2014	Meta-analysis	Preterm birth → higher risk of T1D (OR 1.18) and T2D (OR 1.51).

## Data Availability

No new data were created or analysed in this study. Data sharing is not applicable to this article.

## References

[1] Group CoPoLBI (2023). New World Health Organization recommendations for care of preterm or low birth weight infants: Health policy. EClinicalMedicine.

[2] Lawn J.E., Ohuma E.O., Bradley E., Idueta L.S., Hazel E., Okwaraji Y.B., Erchick D.J., Yargawa J., Katz J., Lee A.C.C. (2023). Small babies, big risks: Global estimates of prevalence and mortality for vulnerable newborns to accelerate change and improve counting. Lancet.

[3] Liang X., Lyu Y., Li J., Li Y., Chi C. (2024). Global, regional, and national burden of preterm birth, 1990-2021: A systematic analysis from the global burden of disease study 2021. EClinicalMedicine.

[4] Perin J., Mulick A., Yeung D., Villavicencio F., Lopez G., Strong K.L., Prieto-Merino D., Cousens S., Black R.E., Liu L. (2022). Global, regional, and national causes of under-5 mortality in 2000-19: An updated systematic analysis with implications for the Sustainable Development Goals. Lancet Child Adolesc. Health.

[5] Cao G., Liu J., Liu M. (2022). Global, Regional, and National Incidence and Mortality of Neonatal Preterm Birth, 1990–2019. JAMA Pediatr..

[6] Venkatesan T., Rees P., Gardiner J., Battersby C., Purkayastha M., Gale C., Sutcliffe A.G. (2023). National Trends in Preterm Infant Mortality in the United States by Race and Socioeconomic Status, 1995–2020. JAMA Pediatr..

[7] Risnes K., Bilsteen J.F., Brown P., Pulakka A., Andersen A.N., Opdahl S., Kajantie E., Sandin S. (2021). Mortality Among Young Adults Born Preterm and Early Term in 4 Nordic Nations. JAMA Netw. Open.

[8] Sun B., Bertolet M., Brooks M.M., Hubel C.A., Lewis C.E., Gunderson E.P., Catov J.M. (2020). Life Course Changes in Cardiometabolic Risk Factors Associated With Preterm Delivery: The 30-Year CARDIA Study. J. Am. Heart Assoc..

[9] de Mendonça E.L.S.S., de Lima Macêna M., Bueno N.B., de Oliveira A.C.M., Mello C.S. (2020). Premature birth, low birth weight, small for gestational age and chronic non-communicable diseases in adult life: A systematic review with meta-analysis. Early Hum. Dev..

[10] Cheong J.L.Y., Haikerwal A., Wark J.D., Irving L., Garland S.M., Patton G.C., Cheung M.M., Doyle L.W., Group V.I.C.S. (2020). Cardiovascular Health Profile at Age 25 Years in Adults Born Extremely Preterm or Extremely Low Birthweight. Hypertension.

[11] (NCD-RisC) N.R.F.C. (2024). Worldwide trends in underweight and obesity from 1990 to 2022: A pooled analysis of 3663 population-representative studies with 222 million children, adolescents, and adults. Lancet.

[12] D’Agata A.L., Green C.E., Sullivan M.C. (2022). A new patient population for adult clinicians: Preterm born adults. Lancet Reg. Health Am..

[13] Raju T.N.K., Buist A.S., Blaisdell C.J., Moxey-Mims M., Saigal S. (2017). Adults born preterm: A review of general health and system-specific outcomes. Acta Paediatr..

[14] Carr H., Cnattingius S., Granath F., Ludvigsson J.F., Edstedt Bonamy A.K. (2017). Preterm Birth and Risk of Heart Failure Up to Early Adulthood. J. Am. Coll. Cardiol..

[15] Kajantie E., Osmond C., Eriksson J.G. (2015). Coronary Heart Disease and Stroke in Adults Born Preterm—The Helsinki Birth Cohort Study. Paediatr. Perinat. Epidemiol..

[16] Crump C., Howell E.A., Stroustrup A., McLaughlin M.A., Sundquist J., Sundquist K. (2019). Association of Preterm Birth With Risk of Ischemic Heart Disease in Adulthood. JAMA Pediatr..

[17] Yang F., Janszky I., Gissler M., Cnattingius S., Roos N., Miao M., Yuan W., Li J., László K.D. (2023). Preterm Birth, Small for Gestational Age, and Large for Gestational Age and the Risk of Atrial Fibrillation Up to Middle Age. JAMA Pediatr..

[18] Crump C., Sundquist J., Sundquist K. (2021). Stroke Risks in Adult Survivors of Preterm Birth: National Cohort and Cosibling Study. Stroke.

[19] Osmond C., Kajantie E., Forsén T.J., Eriksson J.G., Barker D.J. (2007). Infant growth and stroke in adult life: The Helsinki birth cohort study. Stroke.

[20] Ueda P., Cnattingius S., Stephansson O., Ingelsson E., Ludvigsson J.F., Bonamy A.K. (2014). Cerebrovascular and ischemic heart disease in young adults born preterm: A population-based Swedish cohort study. Eur. J. Epidemiol..

[21] Crump C., Sundquist J., Sundquist K. (2020). Preterm birth and risk of type 1 and type 2 diabetes: A national cohort study. Diabetologia.

[22] Kajantie E., Osmond C., Barker D.J., Eriksson J.G. (2010). Preterm birth—A risk factor for type 2 diabetes? The Helsinki birth cohort study. Diabetes Care.

[23] Li S., Zhang M., Tian H., Liu Z., Yin X., Xi B. (2014). Preterm birth and risk of type 1 and type 2 diabetes: Systematic review and meta-analysis. Obes. Rev..

[24] Burchert H., Lewandowski A.J. (2019). Preterm Birth Is a Novel, Independent Risk Factor for Altered Cardiac Remodeling and Early Heart Failure: Is it Time for a New Cardiomyopathy?. Curr. Treat. Options Cardiovasc. Med..

[25] Dlamini N., Jordan L.C. (2021). Young Adult Survivors of Preterm Birth Are at Increased Risk of Stroke: The Missing Link. Stroke.

[26] Lawlor D.A., Ronalds G., Clark H., Smith G.D., Leon D.A. (2005). Birth weight is inversely associated with incident coronary heart disease and stroke among individuals born in the 1950s: Findings from the Aberdeen Children of the 1950s prospective cohort study. Circulation.

[27] Crump C., Sundquist J., Sundquist K. (2025). Adverse pregnancy outcomes and long-term stroke risks. Eur. Heart J..

[28] Goldacre R.R. (2018). Associations between birthweight, gestational age at birth and subsequent type 1 diabetes in children under 12: A retrospective cohort study in England, 1998–2012. Diabetologia.

[29] Crump C. (2020). An overview of adult health outcomes after preterm birth. Early Hum. Dev..

[30] Reisinger C., Nkeh-Chungag B.N., Fredriksen P.M., Goswami N. (2021). The prevalence of pediatric metabolic syndrome-a critical look on the discrepancies between definitions and its clinical importance. Int. J. Obes..

[31] Jańczewska I., Wierzba J., Jańczewska A., Szczurek-Gierczak M., Domżalska-Popadiuk I. (2023). Prematurity and Low Birth Weight and Their Impact on Childhood Growth Patterns and the Risk of Long-Term Cardiovascular Sequelae. Children.

[32] Ou-Yang M.C., Sun Y., Liebowitz M., Chen C.C., Fang M.L., Dai W., Chuang T.W., Chen J.L. (2024). Correction: Accelerated weight gain, prematurity, and the risk of childhood obesity: A meta-analysis and systematic review. PLoS ONE.

[33] Freedman D.S., Khan L.K., Serdula M.K., Dietz W.H., Srinivasan S.R., Berenson G.S. (2005). The relation of childhood BMI to adult adiposity: The Bogalusa Heart Study. Pediatrics.

[34] Freedman D.S., Khan L.K., Serdula M.K., Dietz W.H., Srinivasan S.R., Berenson G.S. (2004). Inter-relationships among childhood BMI, childhood height, and adult obesity: The Bogalusa Heart Study. Int. J. Obes. Relat. Metab. Disord..

[35] Jacobs D.R., Woo J.G., Sinaiko A.R., Daniels S.R., Ikonen J., Juonala M., Kartiosuo N., Lehtimäki T., Magnussen C.G., Viikari J.S.A. (2022). Childhood Cardiovascular Risk Factors and Adult Cardiovascular Events. N. Engl. J. Med..

[36] Barker D.J., Eriksson J.G., Forsén T., Osmond C. (2002). Fetal origins of adult disease: Strength of effects and biological basis. Int. J. Epidemiol..

[37] Reynolds R.M., Jacobsen G.H., Drake A.J. (2013). What is the evidence in humans that DNA methylation changes link events in utero and later life disease?. Clin. Endocrinol..

[38] Qiu J. (2006). Epigenetics: Unfinished symphony. Nature.

[39] Johnson M.J., Wootton S.A., Leaf A.A., Jackson A.A. (2012). Preterm birth and body composition at term equivalent age: A systematic review and meta-analysis. Pediatrics.

[40] Roberts G., Cheong J.L. (2014). Long-term growth and general health for the tiniest or most immature infants. Semin. Fetal Neonatal Med..

[41] Uthaya S., Thomas E.L., Hamilton G., Doré C.J., Bell J., Modi N. (2005). Altered adiposity after extremely preterm birth. Pediatr. Res..

[42] De Lucia Rolfe E., Loos R.J., Druet C., Stolk R.P., Ekelund U., Griffin S.J., Forouhi N.G., Wareham N.J., Ong K.K. (2010). Association between birth weight and visceral fat in adults. Am. J. Clin. Nutr..

[43] Stansfield B.K., Fain M.E., Bhatia J., Gutin B., Nguyen J.T., Pollock N.K. (2016). Nonlinear Relationship between Birth Weight and Visceral Fat in Adolescents. J. Pediatr..

[44] Crane J.D., Yellin S.A., Ong F.J., Singh N.P., Konyer N., Noseworthy M.D., Schmidt L.A., Saigal S., Morrison K.M. (2016). ELBW survivors in early adulthood have higher hepatic, pancreatic and subcutaneous fat. Sci. Rep..

[45] Sipola-Leppänen M., Hovi P., Andersson S., Wehkalampi K., Vääräsmäki M., Strang-Karlsson S., Järvenpää A.L., Mäkitie O., Eriksson J.G., Kajantie E. (2011). Resting energy expenditure in young adults born preterm--the Helsinki study of very low birth weight adults. PLoS ONE.

[46] Huang W., Meir A.Y., Olapeju B., Wang G., Hong X., Venkataramani M., Cheng T.L., Igusa T., Liang L., Wang X. (2023). Defining longitudinal trajectory of body mass index percentile and predicting childhood obesity: Methodologies and findings in the Boston Birth Cohort. Precis. Nutr..

[47] Ni Y., Beckmann J., Hurst J.R., Morris J.K., Marlow N. (2021). Size at birth, growth trajectory in early life, and cardiovascular and metabolic risks in early adulthood: EPICure study. Arch. Dis. Child. Fetal Neonatal Ed..

[48] Ekelund U., Ong K., Linné Y., Neovius M., Brage S., Dunger D.B., Wareham N.J., Rössner S. (2006). Upward weight percentile crossing in infancy and early childhood independently predicts fat mass in young adults: The Stockholm Weight Development Study (SWEDES). Am. J. Clin. Nutr..

[49] Nakano Y. (2020). Adult-Onset Diseases in Low Birth Weight Infants: Association with Adipose Tissue Maldevelopment. J. Atheroscler. Thromb..

[50] Spalding K.L., Arner E., Westermark P.O., Bernard S., Buchholz B.A., Bergmann O., Blomqvist L., Hoffstedt J., Näslund E., Britton T. (2008). Dynamics of fat cell turnover in humans. Nature.

[51] Virtue S., Vidal-Puig A. (2010). Adipose tissue expandability, lipotoxicity and the Metabolic Syndrome—An allostatic perspective. Biochim. Biophys. Acta.

[52] Sbarbati A., Osculati F., Silvagni D., Benati D., Galiè M., Camoglio F.S., Rigotti G., Maffeis C. (2006). Obesity and inflammation: Evidence for an elementary lesion. Pediatrics.

[53] Maffeis C., Silvagni D., Bonadonna R., Grezzani A., Banzato C., Tatò L. (2007). Fat cell size, insulin sensitivity, and inflammation in obese children. J. Pediatr..

[54] Maffeis C., Morandi A. (2018). Body composition and insulin resistance in children. Eur. J. Clin. Nutr..

[55] Hershkop K., Besor O., Santoro N., Pierpont B., Caprio S., Weiss R. (2016). Adipose Insulin Resistance in Obese Adolescents Across the Spectrum of Glucose Tolerance. J. Clin. Endocrinol. Metab..

[56] Valenti L., Bugianesi E., Pajvani U., Targher G. (2016). Nonalcoholic fatty liver disease: Cause or consequence of type 2 diabetes?. Liver Int..

[57] Li Z., Lin M., Liu C., Wang D., Shi X., Chen Z., Liu Y., Yang S., Li X. (2018). Fetuin-B links nonalcoholic fatty liver disease to type 2 diabetes via inducing insulin resistance: Association and path analyses. Cytokine.

[58] Sipola-Leppänen M., Vääräsmäki M., Tikanmäki M., Hovi P., Miettola S., Ruokonen A., Pouta A., Järvelin M.R., Kajantie E. (2014). Cardiovascular risk factors in adolescents born preterm. Pediatrics.

[59] Heikkilä K., Metsälä J., Pulakka A., Nilsen S.M., Kivimäki M., Risnes K., Kajantie E. (2023). Preterm birth and the risk of multimorbidity in adolescence: A multiregister-based cohort study. Lancet Public Health.

[60] Hochmayr C., Ndayisaba J.P., Gande N., Staudt A., Bernar B., Stock K., Kiechl S.J., Geiger R., Griesmaier E., Knoflach M. (2023). Cardiovascular health profiles in adolescents being born term or preterm-results from the EVA-Tyrol study. BMC Cardiovasc. Disord..

[61] Fernandes R.O., Dos Santos V.B., Saalfeld R.M., Evaristo A.S., Bernardi J.R., Rovedder P.M.E., Nuyt A.M., Luu T.M., Procianoy R.S., Silveira R.C. (2025). Cardiometabolic characteristics of school-aged children born preterm with very low birth weight. Pediatr. Res..

[62] Cauzzo C., Chiavaroli V., Di Valerio S., Chiarelli F. (2023). Birth size, growth trajectory and later cardio-metabolic risk. Front. Endocrinol..

[63] Sonaglioni A., Cerini F., Cerrone A., Argiento L., Nicolosi G.L., Rigamonti E., Lombardo M., Rumi M.G., Viganò M. (2022). Liver stiffness measurement identifies subclinical myocardial dysfunction in non-advanced non-alcoholic fatty liver disease patients without overt heart disease. Intern. Emerg. Med..

[64] Hirose K., Nakanishi K., Di Tullio M.R., Homma S., Sawada N., Yoshida Y., Hirokawa M., Koyama K., Kimura K., Nakao T. (2023). Association between non-alcoholic fatty liver disease and subclinical left ventricular dysfunction in the general population. Eur. Heart J. Open.

[65] Kumar V.H.S. (2022). Cardiovascular Morbidities in Adults Born Preterm: Getting to the Heart of the Matter!. Children.

[66] Lewandowski A.J., Levy P.T., Bates M.L., McNamara P.J., Nuyt A.M., Goss K.N. (2020). Impact of the Vulnerable Preterm Heart and Circulation on Adult Cardiovascular Disease Risk. Hypertension.

[67] Puente B.N., Kimura W., Muralidhar S.A., Moon J., Amatruda J.F., Phelps K.L., Grinsfelder D., Rothermel B.A., Chen R., Garcia J.A. (2014). The oxygen-rich postnatal environment induces cardiomyocyte cell-cycle arrest through DNA damage response. Cell.

[68] Lewandowski A.J., Augustine D., Lamata P., Davis E.F., Lazdam M., Francis J., McCormick K., Wilkinson A.R., Singhal A., Lucas A. (2013). Preterm heart in adult life: Cardiovascular magnetic resonance reveals distinct differences in left ventricular mass, geometry, and function. Circulation.

[69] Lewandowski A.J., Bradlow W.M., Augustine D., Davis E.F., Francis J., Singhal A., Lucas A., Neubauer S., McCormick K., Leeson P. (2013). Right ventricular systolic dysfunction in young adults born preterm. Circulation.

[70] Goss K.N., Haraldsdottir K., Beshish A.G., Barton G.P., Watson A.M., Palta M., Chesler N.C., Francois C.J., Wieben O., Eldridge M.W. (2020). Association Between Preterm Birth and Arrested Cardiac Growth in Adolescents and Young Adults. JAMA Cardiol..

[71] Huckstep O.J., Williamson W., Telles F., Burchert H., Bertagnolli M., Herdman C., Arnold L., Smillie R., Mohamed A., Boardman H. (2018). Physiological Stress Elicits Impaired Left Ventricular Function in Preterm-Born Adults. J. Am. Coll. Cardiol..

[72] Bonafiglia E., Garzon S., Forte N., Bosco M., Ciuffreda M., Cristofaletti A., Hoxha S., Beghini R., Uccella S., Gottin L. (2025). Association of patent ductus arteriosus duration with bronchopulmonary dysplasia and mortality: A cohort study. Pediatr. Res..

[73] Ficial B., Corsini I., Clemente M., Cappelleri A., Remaschi G., Quer L., Urbani G., Sandrini C., Biban P., Dani C. (2022). Feasibility, Reproducibility and Reference Ranges of Left Atrial Strain in Preterm and Term Neonates in the First 48 h of Life. Diagnostics.

[74] Huckstep O.J., Burchert H., Williamson W., Telles F., Tan C.M.J., Bertagnolli M., Arnold L., Mohamed A., McCormick K., Hanssen H. (2021). Impaired myocardial reserve underlies reduced exercise capacity and heart rate recovery in preterm-born young adults. Eur. Heart J. Cardiovasc. Imaging.

[75] Goss K.N., Beshish A.G., Barton G.P., Haraldsdottir K., Levin T.S., Tetri L.H., Battiola T.J., Mulchrone A.M., Pegelow D.F., Palta M. (2018). Early Pulmonary Vascular Disease in Young Adults Born Preterm. Am. J. Respir. Crit. Care Med..

[76] Hovi P., Vohr B., Ment L.R., Doyle L.W., McGarvey L., Morrison K.M., Evensen K.A., van der Pal S., Grunau R.E., Brubakk A.M. (2016). Blood Pressure in Young Adults Born at Very Low Birth Weight: Adults Born Preterm International Collaboration. Hypertension.

[77] Bertagnolli M., Luu T.M., Lewandowski A.J., Leeson P., Nuyt A.M. (2016). Preterm Birth and Hypertension: Is There a Link?. Curr. Hypertens. Rep..

[78] Boardman H., Birse K., Davis E.F., Whitworth P., Aggarwal V., Lewandowski A.J., Leeson P. (2016). Comprehensive multi-modality assessment of regional and global arterial structure and function in adults born preterm. Hypertens. Res..

[79] Didion S.P. (2017). A novel genetic model to explore the Brenner hypothesis: Linking nephron endowment and number with hypertension. Med. Hypotheses.

[80] Vidal E., Trevisanuto D. (2025). From survival to surveillance: The long-term kidney legacy of preterm birth. Pediatr. Nephrol..

[81] Sacco A., Cornish E.F., Marlow N., David A.L., Giussani D.A. (2023). The effect of antenatal corticosteroid use on offspring cardiovascular function: A systematic review. BJOG.

